# Immune Evaluation of Recombinant *Lactobacillus plantarum* With Surface Display of HA1-DCpep in Mice

**DOI:** 10.3389/fimmu.2021.800965

**Published:** 2021-12-01

**Authors:** Hui Niu, Jun-Hong Xing, Bo-Shi Zou, Chun-Wei Shi, Hai-Bin Huang, Yan-Long Jiang, Jian-Zhong Wang, Xin Cao, Nan Wang, Yan Zeng, Wen-Tao Yang, Gui-Lian Yang, Chun-Feng Wang

**Affiliations:** College of Veterinary Medicine, College of Animal Science and Technology, Jilin Provincial Engineering Research Center of Animal Probiotics, Jilin Provincial Key Laboratory of Animal Microecology and Healthy Breeding, Key Laboratory of Animal Production and Product Quality Safety of the Ministry of Education, Jilin Agricultural University, Changchun, China

**Keywords:** H7N9 avian influenza, HA1 protein, DCpep, *Lactobacillus plantarum*, immune response

## Abstract

*Avian influenza viruses* can be efficiently transmitted through mucous membranes, and conventional vaccines are not effective in protecting against mucosal infection by influenza viruses. To induce multiple immune responses in an organism, we constructed a recombinant *Lactobacillus plantarum* expressing the influenza virus antigen HA1 with the adjuvant dendritic cell-targeting peptide (DCpep). The recombinant *L. plantarum* strains NC8Δ-pWCF-HA1 and NC8Δ-pWCF-HA1-DCpep were used to immunize mice *via* oral administration, and the humoral, cellular and mucosal immune responses were evaluated. In addition, the serum levels of specific antibodies and hemagglutination inhibition (HI) levels were also measured. Our results showed that recombinant *L. plantarum* activated dendritic cells in Peyer’s patches (PPs), increased the numbers of CD4^+^IFN-γ^+^ and CD8^+^IFN-γ^+^ cells in the spleen and mesenteric lymph nodes (MLNs), and affected the ability of CD4^+^ and CD8^+^ cells to proliferate in the spleen and MLNs. Additionally, recombinant *L. plantarum* increased the number of B220^+^IgA^+^ cells in PPs and the level of IgA in the lungs and different intestinal segments. In addition, specific IgG, IgG1 and IgG2a antibodies were induced at high levels in the mice serum, specific IgA antibodies were induced at high levels in the mice feces, and HI potency was significantly increased. Thus, the recombinant *L. plantarum* strains NC8Δ-pWCF-HA1 and NC8Δ-pWCF-HA1-DCpep have potential as vaccine candidates for avian influenza virus.

## Introduction

Avian influenza viruses (AIVs) have broken the genetic barrier and acquired the ability to directly infect not only birds but also humans, causing substantial economic damage to the poultry industry and posing a serious risk to human health and public health (1). The genome of avian influenza virus contains eight single-stranded negative-sense RNA fragments, and the unique fragmented genomic features make the virus highly susceptible to recombination and often lead to the creation of new viruses (2). The differences in antigenicity of the hemagglutinin (HA) and neuraminidase (NA) protein segments can be divided into 16 subtypes for the HA protein segment and 9 subtypes for the NA protein segment (3). H7N9 subtype avian influenza virus is a highly pathogenic avian influenza virus, first emerged in China in 2013, resulting in 1,568 human infections and causing 615 deaths (4). To date, vaccines based on AIV surface proteins are the most effective means of controlling the spread of AIVs.

Hemagglutinin is an important multifunctional protein in influenza viruses. HA is a trimer with a large globular immunodominant head, and each HA monomer is synthesized as an inactive precursor protein, HA0. The HA0 protein is cleaved into the HA1 and HA2 subunits by cytosolic proteases, and the HA1 subunit, which forms the globular head, is highly variable among isoforms and contains the major neutralizing epitope (5). Kamble et al. demonstrated that live attenuated nutrient-deficient mutants of *Salmonella typhimurium* expressing the immunogenic HA1 protein enhance immunity to H1N1 influenza virus infection (6). In addition, Te Beest et al. demonstrated that anti-HA1 antibody-mediated immune responses are more cross-reactive than previously thought and can result in sustained humoral immunity against influenza A virus (7). However, oral vaccines based on the HA1 protein have rarely been reported.

The function of dendritic cells (DCs) is to take up, process and deliver antigens to stimulate an immune response in the body. These cells are the most powerful antigen-presenting cells, as they mature to recognize and process exogenous antigens and present antigenic peptides to naive T cells to induce T cell activation and proliferation (8). DCs are the initiators of the adaptive immune response and the “bridge” between the innate and adaptive immune systems. Dendritic cell-targeting peptide (DCpep) is composed of 12 amino acids and is strongly targeted to DCs, resulting in a relatively strong immune response (9). Xu et al. demonstrated that DCpep-modified chimeric viruslike particle(cVLP) activated DCs *in vitro* and induced effective immune stimulation in chickens with enhanced secretory immunoglobulin A (sIgA) secretion and splenic T cell differentiation (10). The use of DCpep as an adjuvant, therefore, has the advantage of consisting of short sequences and solves the challenge of label expression, as well as providing ready antigen presentation and further stimulation of the immune system.

Lactic acid bacteria (LAB) is an internationally recognized food-grade microorganism that has been used in many fields to increase beneficial intestinal flora and improve human gastrointestinal tract function and immune regulation (11). Shin et al. showed that *L. plantarum* (LRCC5310), which was shown to inhibit rotavirus adhesion and proliferation in the small intestine in animal studies, improved clinical symptoms (12). Park et al. showed that oral or intranasal administration of *L. plantarum* DK119 to mice modulated innate immunity to provide protection against influenza virus (13). Recombinant exogenous proteins of *L. plantarum* can usually be expressed anchored on the surface of bacterium by targeted transport of plasmid expression vectors. Wang et al. demonstrated that the SARS-CoV-2 spike-in protein can be efficiently expressed on the surface of recombinant *L. plantarum* strains and the expressed protein exhibited high antigenicity (14). Oral administration of HA2-LTB-expressing recombinant *Lactobacillus* strains effectively protects mice from H9N2 subtype AIV and increases T cell expression of SIgA-responsive antibodies (15).

Polyglutamate synthase A (pgsA) is derived from *Bacillus subtilis* and is often used as a surface display element, with examples of successful applications seen in the surface display of recipient strains. In most of our previous studies, vectors with resistance screening markers were used, which had environmental impact (16). In this study, An *E. coli-Lactobacillus* shuttle expression vector was used to construct an intermediate host using the aspartic acid-β-semialdehyde dehydrogenase (asd) gene and the alanine racemase (alr) gene as antibiotic-free screening markers and asd gene-deficient *E. coli* (*E. coli* χ6212) as the plasmid donor. The alr gene deletion *L. plantarum* strain NC8Δ (17) was used as the host strain. We used pWCF as an expression vector to achieve expression of a pgsA’ gene product as an attachment matrix for HA1-DCpep and HA1 on the surface of *L. plantarum*. The aim of this study was to construct a novel recombinant *L. plantarum* and evaluate its stimulation of immune responses in mice.

## Materials and Methods

### Antigens, Virus and Vaccine

The amino acid sequences of the antigenic polypeptides DCpep (18) and HA1 were synthesized by Shanghai ZiYu Biotech Co., Ltd.; the sequences were FYPSYHSTPQRP and KSYKNTRESPAIVVWGIHHS, respectively. A recombinant *avian influenza virus* (H5+H7) trivalent inactivated vaccine was purchased from Harbin Vico Biotechnology Co.

### Preparation of Recombinant *L. plantarum*

To construct recombinant *L. plantarum*, the sequence of the HA1 gene was queried as GenBank: Influenza A virus (A/chicken/China/WYG1/2019(H7N9)) and GenBank: MN700034.1. The HA1 sequence was tandemly linked to 3 DCpep peptides as the target fragment (18). This fragment was synthesized and ligated into the pUC-GW-Kan vector by GENEWIZ. To obtain the HA1 fragment from the pUC-HA1-DCpep plasmid, the primers HF and HR were designed with the sequences HF: 5’-TCTAGAATGGACAAAATCTGCCTCG-3’ and HR: 5’-AAGCTTATCTCGCAGTCCGTTTTCT-3’. The HA1 fragment was obtained by PCR and ligated into the pEASY-Blunt-zero vector. The reaction conditions of PCR are 25 cycles of denaturation at 98°C for 10s, annealing at 65°C for 15s, and extension at 72°C for 10s.The vector was obtained as a spare from the recombinant plasmid 409ata kept in the laboratory. We treated the plasmids with *Xba*I and *Hind*III to obtain the gene fragments HA1 and HA1-DCpep. The gene fragments HA1 and HA1-DCpep were subsequently cloned to the pWCF fragment using T4 ligase to construct the NC8Δ-pWCF-HA1 and pWCF-HA1-DCpep vectors. Next, the pWCF-HA1 and pWCF-HA1-DCpep plasmids were electrotransformed into the *L. plantarum* strain NC8Δ, and the positive recombinant bacteria were identified by restriction endonuclease digestion and named NC8Δ-pWCF-HA1 and NC8Δ-pWCF-HA1-DCpep, respectively.

### Western Blotting

Recombinant *L. plantarum* stored at -80°C were activated, inoculated into 5 mL of MRS liquid medium and incubated overnight at 37°C in an anaerobic workstation. The next day, the bacteria was transferred to 50 mL centrifuge tubes at a 1:40 dilution, and all recombinant *L. plantarum* was incubated at 30°C under anaerobic conditions. SppIp-inducing peptide (50 ng/mL) was added when the OD_600_ reached 0.3. After induction, recombinant *L. plantarum* was collected for ultrasonic fragmentation or deposited at -80°C and subjected to 5 freeze-thaw cycles. Subsequently, SDS–PAGE was performed on 10% acrylamide gels. After transfer to membranes, the membranes were blocked with 5% skim milk powder for 1 h at room temperature. An anti-H7N9 hemagglutinin (SinoBiological, China) mouse monoclonal antibody was incubated overnight at 4°C as the primary antibody, and HRP-labeled goat anti-mouse IgG (Bioss Company, China) was used as the secondary antibody. The incubation was carried out at room temperature for 1.5 hours. Finally, color development was performed using a chemiluminescence enhancement kit (Thermo Scientific, USA).

### Flow Cytometry Assay

Recombinant *L. plantarum* was cultured and induced as described previously (19). A total of 1 x 10^6^ CFU of bacteria were washed, followed by the addition of 1 mL of 1% BSA in PBS and incubation for 1 h. An anti-H7N9 hemagglutinin mouse monoclonal antibody was incubated with the bacteria overnight at 4°C. The samples were washed three times with 1 mL of PBS containing 0.2% Tween-20 and incubated for 1.5 h under light-protected conditions with a PE-labeled anti-mouse secondary antibody (BioLegend, USA); after washing, the samples were examined by flow cytometry (BD LSRFortessa™, USA).

### Immunofluorescence Detection

Recombinant *L. plantarum* expressing DCpep were identified by immunofluorescence. A DCpep-specific polyclonal antibody was obtained by immunizing rabbits with a short DCpep peptide. Briefly, in the 2nd and 4th weeks, the short peptides and Freund’s adjuvant were mixed to immunize the rabbits. Then, in the 5th week, blood was collected from the heart, and serum was isolated to obtain DCpep-specific polyclonal antibodies. The bacterial solution was incubated with a primary antibody, the above-prepared DCpep-specific polyclonal antibody (1:300), and then a secondary antibody, a FITC-labeled goat anti-rabbit antibody (1:800) (BioLegend, USA). Ten microliters of bacterial solution was placed on a glass slide, covered with an anti-fluorescence attenuator (Solarbio, China), and observed under a microscope (Leica Microsystems, Germany) in the dark.

### Animals, Ethics Statement and Experimental Design

Fifty specific pathogen-free (SPF) 6-week-old C57BL/6J mice were provided by Henan Skbex Biotechnology Co. Sterile water and feed were provided at the Experimental Animal Centre of Jilin Agricultural University (JLAU20210423001). All animal experiments were monitored by the Animal Protection and Ethics Committee of Jilin Agricultural University. Mice were randomly divided into PBS, NC8Δ-pWCF, NC8Δ-pWCF-HA1, NC8Δ-pWCF-HA1-DCpep and vaccine groups, for a total of 5 groups with 10 animals per group. A volume of induced recombinant *L. plantarum* was washed 3 times with sterile PBS, resuspended in 200 μL PBS and administered to mice by gavage. Two hundred microliters of PBS was used for gavage in the PBS group, and 100 μL of H5-H7 trivalent inactivated vaccine was administered intramuscularly in the vaccine group. The initial immunizations were performed on Days 1, 3 and 5, and booster immunizations were given on Days 15, 17 and 19. The status of the mice was observed after the booster immunizations, and flow cytometry was performed on Day 29.

### Flow Cytometry

Ten days after booster immunization, three mice from each group were euthanized. Spleens, mesenteric lymph nodes (MLNs) and Peyer’s patches (PPs) were aseptically removed, and single-cell suspensions were prepared. Flow cytometry was performed as previously described for this laboratory (20). A prepared 100-μL PPs cell suspension (1 × 10^6^ cells) was mixed with anti-CD16/CD32 (BD Biosciences, USA) at 4°C, incubated for 5 min, and then directly spiked with anti-CD11c-APC (BD Biosciences, USA), anti-CD80-FITC (BD Biosciences, USA), anti-CD86-PE-Cy7 (BD Biosciences, USA), and anti-MHC-II-PerCP-Cy5.5 (BD Biosciences, USA). The antibodies were incubated for 20 min at 4°C in the dark, and then the cell suspension was washed. The cells were then fixed, permeabilized, centrifuged twice, incubated with IgA-FITC (BD Biosciences, USA) for 20 min at 4°C, washed and passed through a nylon sieve.

Each group of prepared splenocyte and MLNs cell suspensions (150 μL, 1.5×10^6^ cells) was transferred into 24-well cell culture plates, and phorbol-12-myristate-13-acetate (PMA) (Sigma-Aldrich, USA) was added. Only the HA1 peptide was added to the NC8∆-pWCF-HA1 group, while the HA1 and DCpep peptides were added to the rest of the groups and incubated for 8 hours. Protein transport inhibitor (BD Biosciences, USA) was then added and incubated for 4 hours. The cells were washed and blocked by adding anti-CD16/CD32 Pure (5μ), followed by antibody staining using 10 μL each of anti-CD3-PerCP-Cy5.5 (BD Biosciences, USA), anti-CD4-FITC (BD Biosciences, USA) and anti-CD8-APC-Cy7 (BD Biosciences, USA). The cells were then fixed, permeabilized and centrifuged twice, and 10 μL of anti-IFN-γ-APC (BD Biosciences, USA) was added for 20 min at 4°C in the dark. The cells were then washed and passed through a nylon sieve prior to detection. Cells were analyzed using BD fluorescence-activated cell sorting (FACS) on an LSRFortessa™ cell analyzer (BD Biosciences, USA). All data were analyzed using FlowJo 7.6.2 software.

### T Cell Proliferation Assay

Each group of splenocytes and MLNs cells were stained with carboxyfluorescein succinimidyl amino ester (CFSE) in 300 μL (3 × 10^6^ cells), incubated at 37°C for 10 min, and then washed. Two hundred microliters (5.0 mL × 10^5^ cells) was transferred to a 96-well cell culture plate, and PMA and antigenic peptides were added and incubated in a cell culture incubator at 37°C for 3 days. The cells were washed, mixed with anti-CD16/CD32 and blocked for 5 min. Ten microliters each of anti-CD4-PerCP (BD Biosciences, USA) and anti-CD8-APC (BD Biosciences, USA) were added and incubated for 20 min at 4°C in the dark. After washing, the cells were washed through a nylon sieve and ready for testing (LSRFortessa™ flow cytometer, BD Biosciences, USA).

### Enzyme-Linked Immunosorbent Assay

Enzyme-linked immunosorbent assays (ELISAs) were performed as described previously to detect the expression levels of specific IgG, IgG1 and IgG2a in the sera of mice at weeks 2, 4 and 10 and IgA in the feces of mice at weeks 2 and 4 (21). Briefly, 96-well ELISA plates were coated with peptides (100 μL) overnight at 4°C and washed 3 times with PBST, and then 100 μL of PBS blocking solution containing 2% BSA was added to each well and incubated in a 37°C incubator for 2 h. PBST was used to wash the plate 3 times, mice serum (100-fold dilution) and fecal supernatant (10-fold dilution) samples were taken and added to each well (100 μL), and standard curve wells were prepared. Specifically, 1 μg/mL unlabeled purified mouse IgG/IgG1/IgG2a/IgA antibody (Southern Biotech, USA) in 100 μL was added to the first well, followed by the preparation of 2-fold dilutions, and the plates were incubated at 37°C for 2 h. The wells were washed 3 times with PBST. Goat anti-mouse IgG1/IgG2a/IgA alpha chain-HRP (HRP, 10,000-fold dilution) (Southern Biotech, USA) and rabbit anti-mouse IgG (HRP, 1000-fold dilution) (Abcam, UK) in a PBS solution containing 1% BSA were added. The reaction was terminated by adding 50 μL of H_2_SO_4_ termination solution at a concentration of 2 M to each well, and the reaction was incubated for 1 h at 37°C. The plates were washed 3 times with PBST, and TMB (Sigma-Aldrich, USA) was added (100 μL per well) for 10 min at room temperature in the dark. The OD_450_ nm values were read and recorded using an enzymatic marker.

### B Cell Response in Tissue

Mice lungs, duodenums, jejunums and ileums were harvested, fixed in 4% paraformaldehyde and then paraffin embedded after dehydration through an alcohol series and clearing. Sections for immunofluorescence staining were 2 μm thick and subsequently dewaxed using xylene and a graded alcohol series. The sections were heated in 1× antigen repair solution for 20 min. The sections were slowly cooled at room temperature, and the antigen repair solution was discarded. The sections were covered with PBS solution and washed 3 times. Tissues labeled with an immunohistochemistry pen were blocked with 5% BSA and 0.3% Triton-100 PBS for 1 h. After removing the blocking buffer by blotting, anti-B220-APC (250× dilution) (BD Biosciences, USA) and IgA-FITC (200× dilution) (BD Biosciences, USA) were added dropwise to the labeled tissues and stored flat overnight at 4°C in a wet box protected from light. The sections were then soaked three times in PBS solution for 5 min each time. The PBS section was removed by blotting the sections dry, a 4’,6-diamidino-2-phenylindole (DAPI) staining solution was added dropwise and placed flat at room temperature protected from light for 15 min. The PBS solution was used to wash the sections three times, and then any residual PBS was removed by blotting. An anti-fluorescence attenuation sealant was added dropwise, coverslips were mounted, and the sections were observed under a fluorescence microscope (Leica Microsystems, Germany).

### Detection of Hemagglutination Inhibition (HI) Levels in Mice Serum

Blood was collected from mice on Days 0, 14, 28 and 70, and serum was isolated, added to 4 times the volume of receptor-disrupting enzyme (RDE) (Denka Seiken, Japan) and incubated in a 37°C incubator overnight. Then, the serum was inactivated in a 56°C water bath for 50 min and stored at 4°C. The HA test was performed by adding 25 µL of H7 antigen to each well and 25 µL of H7 antigen to the first well; the contents were then diluted in multiples to the 11th well, with 25 µL aspirated and discarded while another 25 μL of PBS was added to each well, and finally 25 µL of 1% chicken red blood cell suspension was added to each well, followed by shaking for 2 min and incubation for 30 min at room temperature. The highest dilution at which no flow was detected when the reaction plate was tilted to 60 degrees was considered the hemagglutination potency of the H7 antigen. The potency of the H7 antigen was divided by 4 to obtain a dilution containing 4 units of the H7 antigen. PBS (25 µL) was added to wells 1 to 11 of the 96-well plate, and 50 µL of PBS was added to well 12. Treated serum (25 µL) was added to well 1 and mixed well, and then 25 µL was moved to well 2. The samples were then double diluted to well 10, and 25 µL was aspirated from well 10 and discarded. The 4HAU antigen (25 µL) was added to wells 1 to 11 and allowed to stand at room temperature for 30 min. Finally, 25 µL of 1% chicken red blood cell suspension was added to each well, shaken to mix, and allowed to stand at room temperature for 30 min. The result was determined when the red blood cells in the control wells were clearly clumped at the bottom of the wells, and the highest dilution of serum that completely inhibited the agglutination of the 4HAU antigen was considered the HI potency of the serum sample.

### Statistical Analysis

Flow cytograms were analyzed using FlowJo 7.6.2 software. GraphPad Prism software was applied to make graphs and perform statistical analyses, and differences among groups were analyzed by one-way ANOVA (*, P < 0.05; **, P < 0.01; ***, P < 0.001; ****, P < 0.0001).

## Results

### Synthesis of pWCF-HA1 and pWCF-HA1-DCpep Expressed on *L. plantarum*

**Figure 1:**
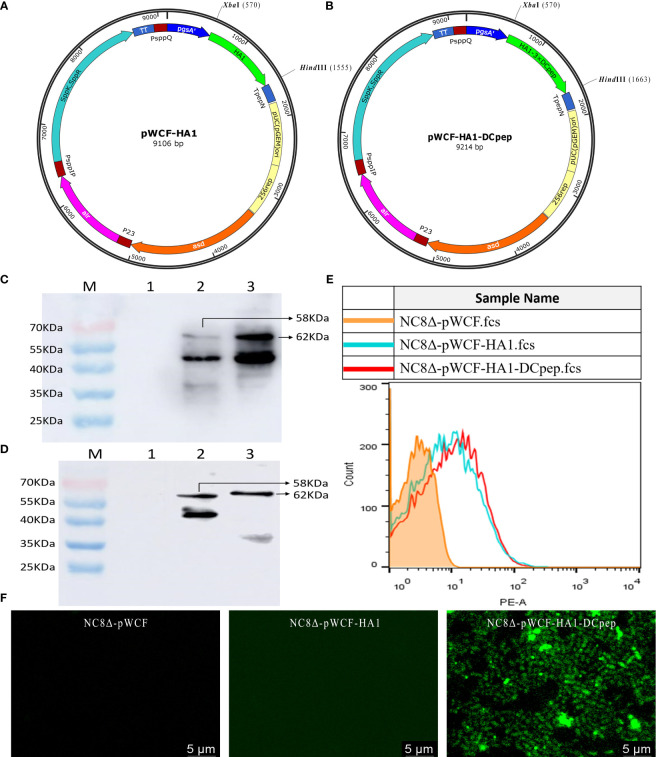
Synthesis of pWCF-HA1 and pWCF-HA1-DCpep on *L. plantarum.* **(A)** Recombinan pWCF-HA1 plasmid mapping. **(B)** Plasmid profile of recombinant pWCF-HA1-DCpep. **(C)** Recombinant bacteria were sonicated, and the synthesis of fusion antigens was detected by immunoblotting using an anti-H7N9 hemagglutinin antibody. M: marker; Lane 1: NC8Δ-pWCF; Lane 2: NC8Δ-pWCF-HA1; Lane 3: NC8Δ-pWCF-HA1-DCpep. **(D)** Recombinant *L. plantarum* were subjected to repeated freeze-thaw cycles, and fusion antigen synthesis was detected by immunoblotting using an anti-H7N9 hemagglutinin antibody M: marker; Lane 1: NC8Δ-pWCF; Lane 2: NC8Δ-pWCF-HA1; Lane 3: NC8Δ-pWCF-HA1-DCpep. **(E)** Flow cytometry with an anti-H7N9 hemagglutinin antibody and PE-conjugated anti-mouse IgG antibody. **(F)** Indirect immunofluorescence analysis with an anti-DCpep antibody followed by indirect immunofluorescence analysis with a FITC-conjugated anti-mouse IgG antibody.

Recombinant pWCF-HA1 and pWCF-HA1-DCpep plasmids expressing influenza virus HA1 were successfully constructed (**Figures 1A, B**). As assessed by immunoblotting using an anti-H7N9 hemagglutinin antibody, positive bands were observed with both ultrasonic fragmentation (**Figure 1C**) and repeated freeze-thaw (**Figure 1D**) methods, but no bands were observed in control samples. To further determine whether HA1 and HA1-DCpep are localized on the surface of *L. plantarum*, detection was performed by flow cytometry and immunofluorescence staining. The results showed that positive fluorescence signals were detected for both NC8Δ-pWCF-HA1 and NC8Δ-pWCF-HA1-DCpep samples compared to control samples (**Figures 1E, F**). The above results indicate that the pWCF-HA1 and pWCF-HA1-DCpep plasmids were successfully constructed and expressed in *L. plantarum*.

### Activation of DC Costimulatory Molecules by *L. plantarum* Expressing HA1 and HA1-DCpep

**Figure 2:**
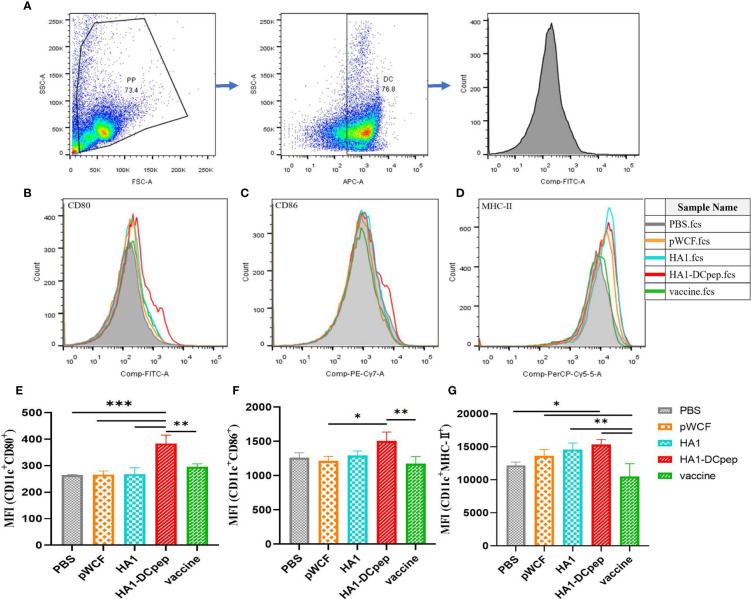
Activation of DC costimulatory molecules by *L. plantarum* expressing HA1 and HA1-DCpep. After booster immunization, mice PPs were harvested, and cell suspensions was prepared using 1 ×10^6^ total cells treated with anti-CD16/CD32 for molecular staining of the DC surface **(A)** Gating strategy for DCs in PPs. Flow cytometry was performed to detect the expression levels of CD80 **(B, E)**, CD86 **(C, F)**, and MHC-II **(D, G)** in DCs in PPs. Statistical significance was assessed by one-way ANOVA (n = 3 mice per group). *P < 0.05; **P < 0.01; ***P < 0.001.

To investigate the effect of NC8Δ-pWCF-HA1 and NC8Δ-pWCF-HA1-DCpep on DCs in PPs of mice, we examined the expression of the activation markers CD80, CD86 and MHC-II on the surface of DCs. The gating method for DCs in PPs is shown in **Figure 2A**. The results showed that the differences in the MFI (CD11c^+^ CD80^+^ MFI) of the PPs between the HA1-DCpep group and the PBS, pWCF and HA1-DCpep groups were highly significant (P < 0.001) and that between the HA1-DCpep group and vaccine group was highly significant (P < 0.01) (**Figures 2B, E**). The difference in the MFI (CD11c^+^ CD86^+^) of the PPs between the HA1-DCpep group and vaccine group was highly significant (P < 0.01), and that between the HA1-DCpep group and the pWCF group was also highly significant (P < 0.05) (**Figures 2C, F**). The MFI (CD11c^+^ MHC-II^+^) of the PPs in the HA1-DCpep group was significantly different (P < 0.05) from that in the PBS group and highly significantly different (P < 0.01) from that in the vaccine group, and the HA1 group was highly significantly different (P < 0.01) from the vaccine group (**Figures 2D, G**). The above results indicate that recombinant *L. plantarum* have an activating effect on DCs in mice PPs.

### Recombinant *L. plantarum* Affects the T Cell Response

**Figure 3:**
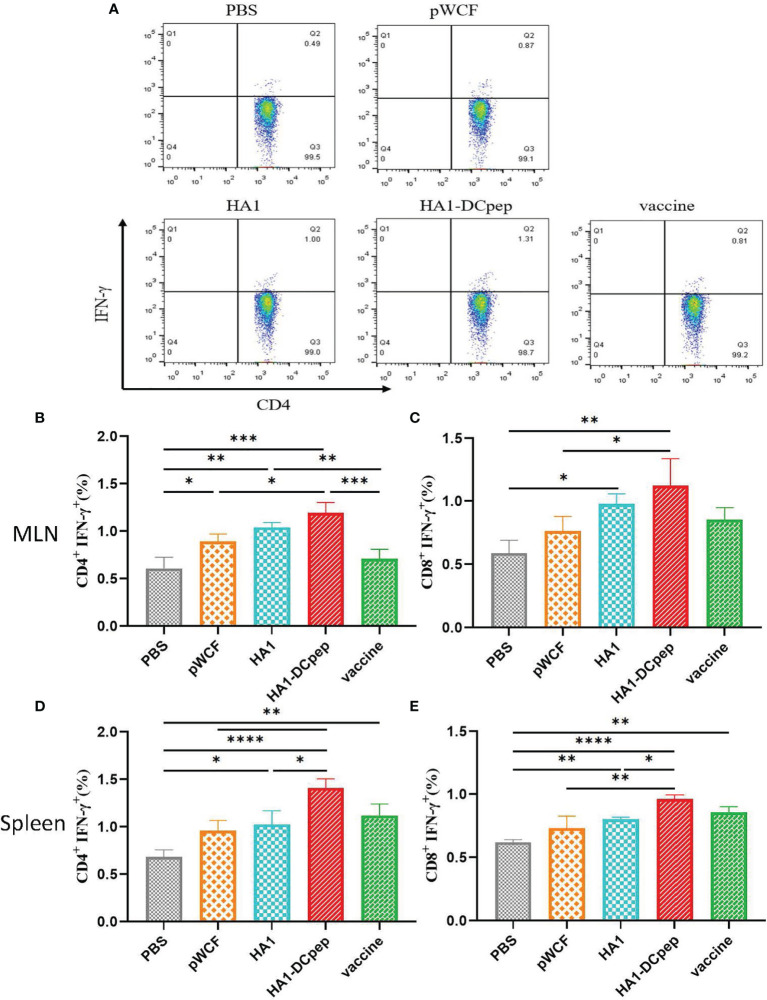
Effect of recombinant *L. plantarum* on T cell responses. After booster immunization, mice MLNs and spleen were collected, and cell suspensions was prepared using a total of 1.5 ×10^6^ cells for plates containing PMA and specific antigenic peptides, and the plates were incubates for a total of 8 h. Scatter plots of CD4^+^IFN-γ^+^ T cells in MLNs in different groups are displayed **(A)**. The numbers of CD4^+^IFN-γ^+^ T cells **(B)** and CD8^+^IFN-γ^+^ T cells **(C)** in the MLNs were detected by flow cytometry after antibody staining, and the numbers of CD4^+^IFN-γ^+^ T cells **(D)** and CD8^+^IFN-γ^+^ T cells **(E)** in the spleen were determined. Statistical signicance was assessed by one-way ANOVA (n = 3 mice per group). *P < 0.05; **P < 0.01; ***P < 0.001; ****P < 0.0001.

Following booster immunization, CD4^+^IFN-γ^+^ and CD8^+^IFN-γ^+^ cells in the MLNs and spleen of mice were examined using flow cytometry, which showed that both the MLN and splenic cellular immune responses of mice could be activated by recombinant *L. plantarum*. A scatter plot of CD4^+^IFN-γ^+^ T cells in the MLNs is displayed (**Figure 3A**), and the full scatter plots are shown in **Figure S1**. CD4^+^IFN-γ^+^ cells were detected in the MLNs of mice. The results showed that the proportion of CD4^+^IFN-γ^+^ cells in the recombinant *L. plantarum* group after oral administration of NC8Δ-pWCF-HA1-DCpep was highly significantly increased compared with that in the PBS and vaccine groups (P < 0.001), and the difference with the pWCF group was significant (P < 0.05). Additionally, among the groups, the HA1-DCpep group had the highest proportion of CD4^+^IFN-γ^+^ cells; the differences between the HA1 group and the pWCF and vaccine groups were highly significant (P < 0.01) (**Figure 3B**). In contrast, when CD8^+^IFN-γ^+^ cells were detected, the proportion of CD8^+^IFN-γ^+^ cells in the orally administered NC8Δ-pWCF-HA1-DCpep recombinant *L. plantarum* group was highly significantly increased (P < 0.01) compared to that in the PBS control group and significantly increased (P < 0.05) compared to that in NC8Δ-pWCF; the difference between the HA1 group and the PBS group was significant (P < 0.05) (**Figure 3C**). CD4^+^IFN-γ^+^ cells in the mice spleen were examined, and the proportion of CD4^+^IFN-γ^+^ cells was highly significantly increased in the NC8Δ-pWCF-HA1-DCpep recombinant *L. plantarum* group after oral administration compared with the PBS control group (P <0.0001), highly significantly increased compared with the pWCF group (P < 0.01), and significantly increased compared with the HA1 group (P < 0.05). Additionally, among the groups, the HA1-DCpep group had the highest proportion of CD4^+^IFN-γ^+^ cells; the difference between the HA1 group and the PBS group was significant (P < 0.05) (**Figure 3D**). In contrast, when CD8^+^IFN-γ^+^ cells were detected, the proportion of CD8^+^IFN-γ^+^ cells was highly significantly different (P < 0.0001) in the orally administered NC8Δ-pWCF-HA1-DCpep recombinant *L. plantarum* group compared to the PBS control group, highly significantly different (P < 0.01) compared to the pWCF group and significantly different (P < 0.05) compared to the HA1 group. Additionally, the HA1-DCpep group had the highest proportion of CD8^+^IFN-γ^+^ cells among the groups; the difference between the HA1 group and the PBS group was highly significant (P < 0.01) (**Figure 3E**).

### Effect of Recombinant *L. plantarum* on T Cell Proliferation

**Figure 4:**
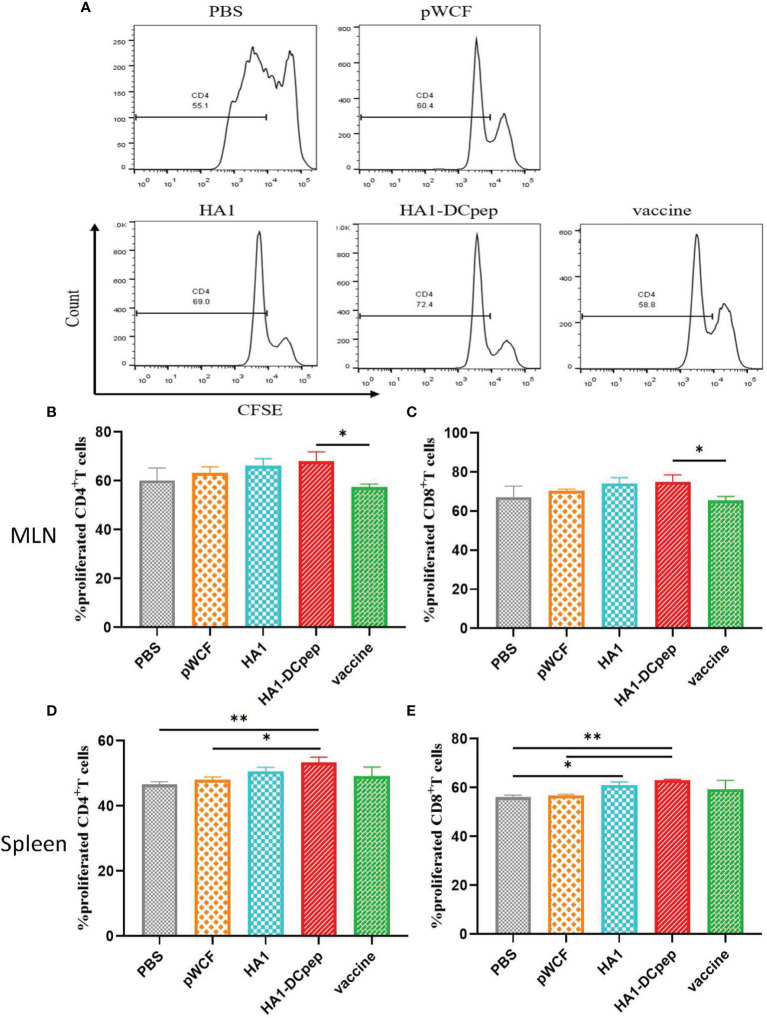
Effect of recombinant *L. plantarum* on T cell proliferation. After booster immunization, mice MLNs cells and splenocytes were stained with CFSE and subsequently cocultured in 96-well U-bottom plates with specific antigenic peptides for 3 days. The number of CD4^+^IFN-γ^+^ T cells in the MLNs in different groups is displayed **(A)**. The proliferation of CD4^+^ T cells **(B)** and CD8^+^ T cells **(C)** in the mice MLNs and that of CD4^+^ T cells **(D)** and CD8^+^ T cells **(E)** in the spleen were examined. Statistical significance was assessed by one-way ANOVA (n = 3 mice per group). *P < 0.05; **P < 0.01.

After booster immunization, T cell proliferation in the mice MLNs and spleen was examined using flow cytometry, and the results showed that both MLNs and splenic T cell proliferation in mice could be activated by recombinant *L. plantarum*. The number of CD4^+^IFN-γ^+^ T cells in the MLNs is displayed (**Figure 4A**), and the full figures are shown in **Figure S2**. For the assay assessing CD4^+^ T cell proliferation in the MLNs in mice, the results showed that the proliferation of CD4^+^ T cells was significantly (P < 0.05) increased in the recombinant *L. plantarum* group after oral administration of NC8Δ-pWCF-HA1-DCpep compared to the vaccine control group (**Figure 4B**). In contrast, when CD8^+^ T cell proliferation was detected, the proliferation of CD8^+^ T cells was significantly (P < 0.05) increased in the orally administered NC8Δ-pWCF-HA1-DCpep recombinant *L. plantarum* group compared to the vaccine control group (**Figure 4C**). CD4^+^ T cell proliferation was examined in the mice spleen. The results showed that CD4^+^ T cell proliferation in the orally administered NC8Δ-pWCF-HA1-DCpep recombinant *L. plantarum* group was highly significantly increased compared to that in the PBS control group (P < 0.01) and significantly increased compared to that in the pWCF group (P < 0.05), and the HA1-DCpep group had the highest CD4^+^ T cell proliferation among all groups (**Figure 4D**). In contrast, for the CD8^+^ T cell proliferation assay, enhancement of the proliferation of CD8^+^ T cells in the recombinant *L. plantarum* group induced *via* oral administration of NC8Δ-pWCF-HA1-DCpep was highly significantly increased (P < 0.01) compared to that in the PBS and pWCF groups; the difference in proliferation between the HA1 group and the pWCF group was significant (P < 0.05) (**Figure 4E**).

### Effect of Recombinant *L. plantarum* on B Cells

**Figure 5:**
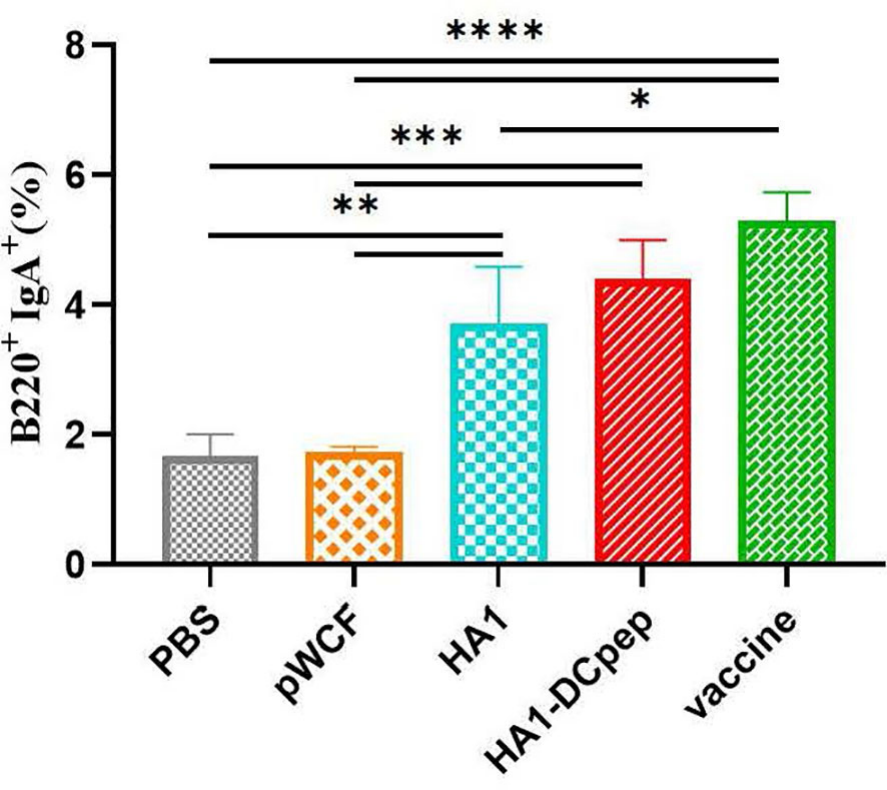
Effect of recombinant *L. plantarum* on B cells. Mice PPs were harvested and used to generate cell suspensions after booster immunization; the cell suspensions were treated with anti-CD16/CD32 and staines with anti-B220 and IgA. Subsequently, the cells were evaluated by flow cytometry. Statistical significance was assessed by one-way ANOVA (n = 3 mice per group). *P < 0.05; **P < 0.01; ***P < 0.001; ****P < 0.0001.

We also investigated whether recombinant *L. plantarum* can induce B cell activation in mice by examining B cells in the PPs of mice after booster immunization. The HA1-DCpep group showed a highly significant increase in the percentage of B220^+^IgA^+^ cells compared to the PBS control and pWCF groups (P < 0.001), while the HA1 group showed a highly significant increase in the percentage of B220^+^IgA^+^ cells compared to the PBS control and pWCF groups (P < 0.01). Additionally, a highly significant increase in the percentage of B220^+^IgA^+^ cells was observed in the vaccine group compared to the PBS control and pWCF groups (P < 0.0001), and a significant increase in the percentage of B220^+^IgA^+^ cells was observed in the vaccine group compared to the HA1 group (P < 0.05) (**Figure 5**).

### Recombinant *L. plantarum* Increase Specific Antibody Levels

**Figure 6:**
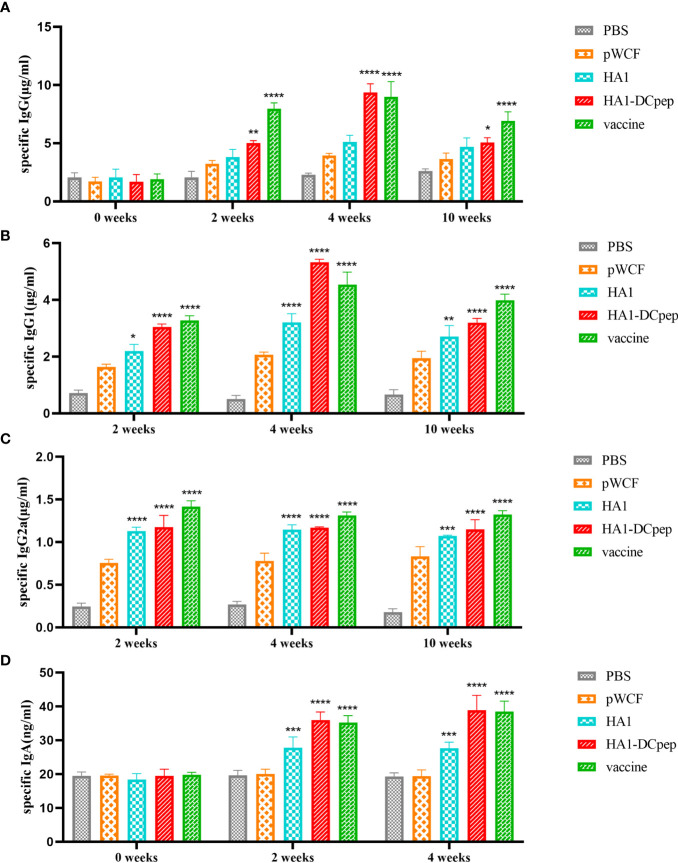
Recombinant *L. plantarum* increase specific antibody levels. The expression levels of specific IgG antibodies in the serum at weeks 0 (before immunization), 2 (2 weeks after primary immunization), 4 (2 weeks after booster immunization) and 10 (10 weeks after primary immunization), **(A)** IgG1 **(B)** and IgG2a **(C)** antibodies in the serum at weeks 2, 4 and 10 and IgA **(D)** antibodies in the feces of mice at weeks 0, 2 and 4 were measured by ELISA. In the same period, HA1, HA1-DCpep and vaccine groups were compared with the pWCF “empty vector” group. Statistical significance was assessed by two-way ANOVA (n = 3 mice per group). *P < 0.05; **P < 0.01; ***P < 0.001; ****P < 0.0001.

To investigate whether recombinant *L. plantarum* can stimulate the body’s immune system and thus induce the production of specific antibodies, we measured IgG in mice serum at weeks 0 (before immunization), 2 (2 weeks after primary immunization), 4 (2 weeks after booster immunization) and 10 (10 weeks after primary immunization). Meanwhile, we also measured IgG1 and IgG2a expression in mice serum at weeks 2, 4 and 10 and IgA expression in mice feces at weeks 0, 2 and 4 using ELISA. We found that IgG expression in the serum was significantly elevated in the HA1-DCpep group compared with the pWCF groups at 2, 4 and 10 weeks (P < 0.0001) (**Figure 6A**), while IgG1 showed similar expression levels in the HA1-DCpep and HA1 groups. In addition, the mice in the HA1-DCpep group produced more IgG1 than those in the HA1 group (**Figure 6B**). Similarly, the expression levels of IgG2a in the serum were significantly elevated in the HA1-DCpep group *versus* the HA1 group at weeks 2, 4 and 10 (**Figure 6C**). Additionally, IgA in the feces of mice was detected. We found that compared with those in the pWCF groups, the IgA expression levels of the HA1-DCpep group, the HA1 group and the vaccine group were surprisingly increased (P<0.0001), and the IgA expression level of the HA1-DCpep group was higher than that of the HA1 group (**Figure 6D**). The results suggest that oral administration of recombinant *L. plantarum* to mice can stimulate the body’s immune response, resulting in the production of specific antibodies.

### Elevated IgA Expression in the Mice Lungs, Duodenum, Jejunum and Ileum

**Figure 7:**
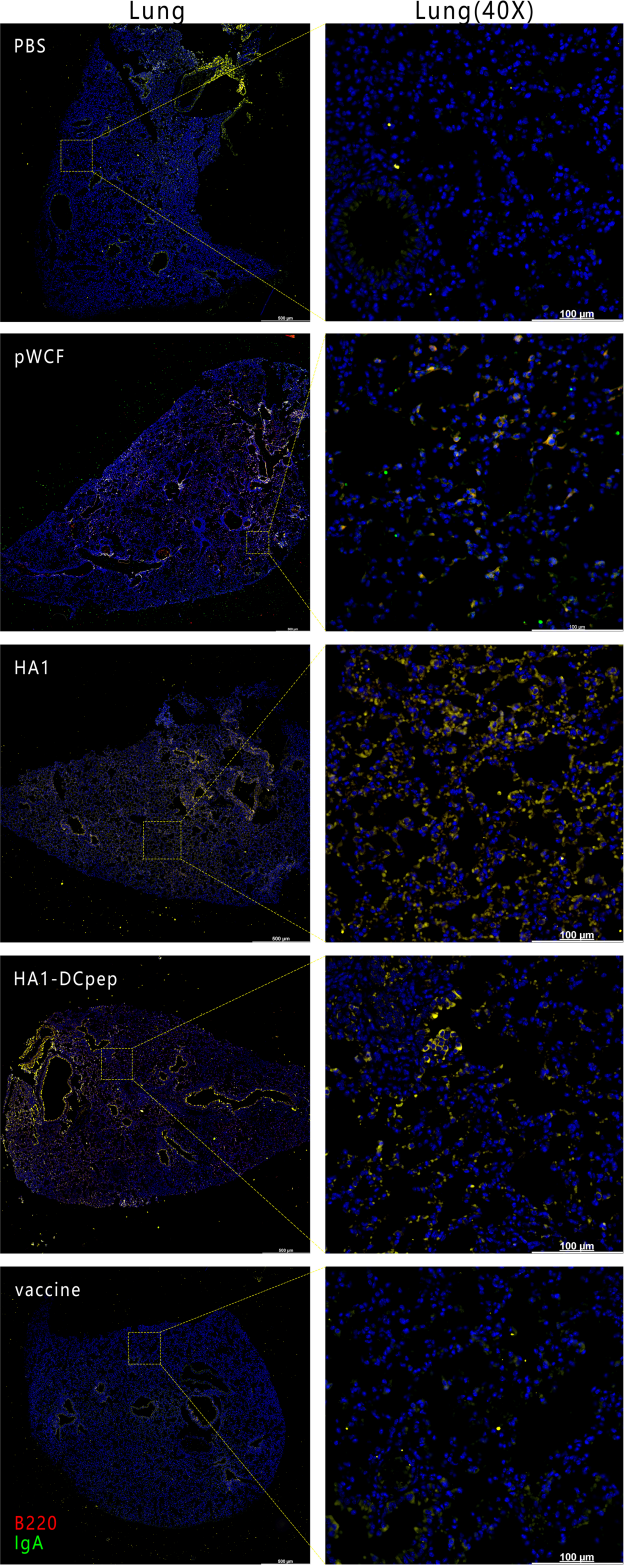
Recombinant *L. plantarum* increase IgA expression in the lungs of mice. After oral immunization of mice with recombinant *L. plantarum*, IgA expression was measured in the lungs using anti-B220 (red) and IgA (green) antibody staining and immunofluorescence techniques. B220^+^IgA^+^ cells were stained yellow. Lung scale bars represent 500 μm (left) and 100 μm (right).

**Figure 8:**
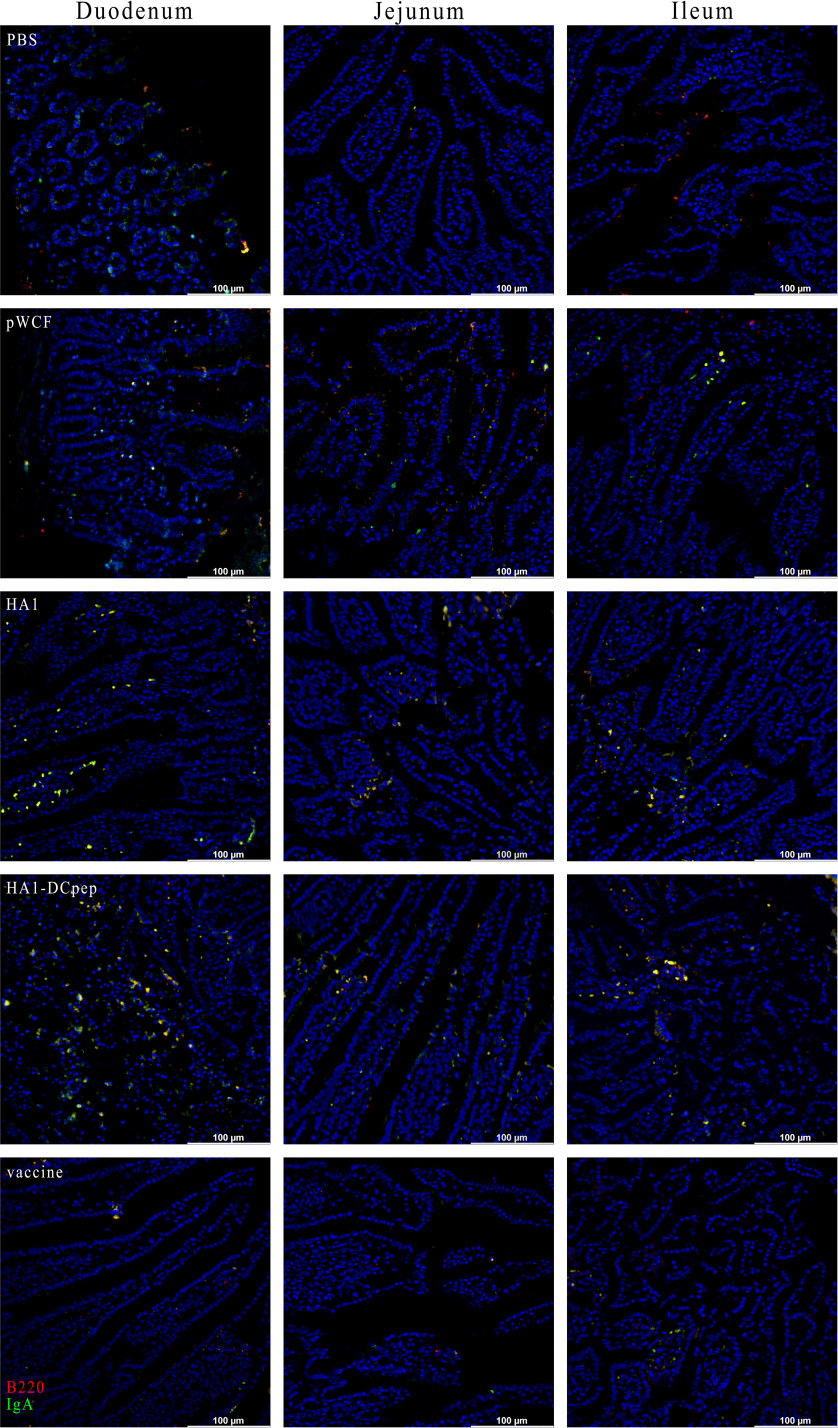
Recombinant *L. plantarum* increase IgA expression in the intestine of mice. After oral immunization of mice with recombinant *L. plantarum*, IgA expression was measured in different sections (duodenum, jejunum, and ileum) using anti-B220 (red) and IgA (green) antibody staining and immunofluorescence technique. Intestinal segment scale bars represent 100 μm.

To verify the expression of IgA induced by recombinant *L. plantarum* expressing the H7N9 antigen at different sites, we measured the expression of IgA in the lungs, duodenum, jejunum and ileum of mice after booster immunization using immunofluorescence staining. The results showed that for the lungs of mice, the HA1 and HA1-DCpep groups exhibited more intense IgA expression than the pWCF group and this expression was even higher than that of the vaccine group (**Figure 7**). Furthermore, the mice in the HA1-DCpep group had the highest IgA expression in the duodenum, jejunum and ileum, while the HA1 group exhibited higher IgA expression in the duodenum than the pWCF group and the vaccine group. The vaccine group produced less IgA in any intestinal segment than the recombinant *L. plantarum* group (**Figure 8**). Thus, recombinant *L. plantarum* were able to effectively induce IgA expression in the lungs and intestine, with the HA1-DCpep group showing a more pronounced advantage.

### Recombinant *L. plantarum* Enhances Serum HI Levels

**Figure 9:**
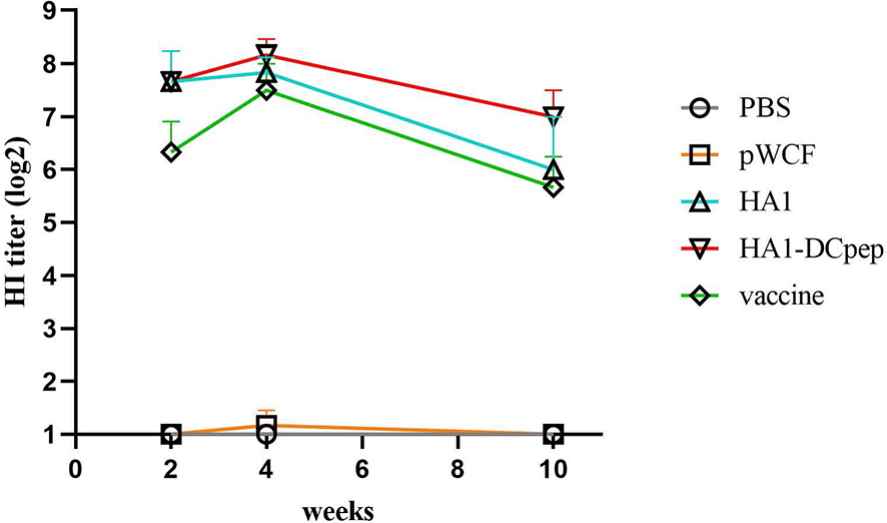
Recombinant Lactobacillus enhances serum HI levels. HI levels were measured in the serum of mice at weeks 2, 4 and 10 after oral immunozation with recombinant *L. plantarum*.

Following immunization with recombinant *L. plantarum*, we measured HI levels in mice sera at weeks 2, 4 and 10 and showed that the HA1-DCpep, HA1 and vaccine groups exhibited higher HI levels at weeks 2, 4 and 10 than the pWCF group, with the HA1-DCpep group showing the highest potency and peaking at week 4. Interestingly, the vaccine group did not show an advantage in HI potency compared to the recombinant *L. plantarum* group immunized with the critical antigen of the linked influenza virus, peaking only at week 4 (**Figure 9**).

## Discussion

Mutations in the neuraminidase and hemagglutinin proteins on the surface of avian influenza virus allow the virus to cross the genus barrier and gain the ability to bind to human receptors, resulting in an interpersonal transmission epidemic (22). Human infections have been detected for avian influenza virus subtypes H5N1, H9N2, H7N9, H10N8, H5N6 and H6N1. Among them, H7N9 infection causes high numbers of severe human cases, causing serious damage to human health (23). Prophylactic vaccination against AIV helps in disease’s prevention in animals, and different vaccination strategies are often used to combat AIV infection (24). The selection of a broad-spectrum, effective vaccine that can be produced quickly and cost-effectively during a pandemic outbreak has become critical. In recent years, recombinant *Lactobacillus* based vaccines have gained advantages in terms of immune efficacy and biological safety (25). In addition, recombinan *Lactobacillus* vaccines can be utilized with adjuvants to enhance antigenic potency, maximize immunogenicity and induce a broad immune response against influenza virus (26).

Recombinant *L. plantarum* expressing different antigens is protective against influenza virus infection. Our previous study applied the resistance screening marker vector NC8-pSIP409-pgsA’ for the expression of antigens (27). The disadvantage of this screening method is that it causes some environmental contamination, whereas in this study, we applied antibiotic-free screening using the alr gene deletion strain NC8Δ as the expressing bacterium, which automatically degrades when the recombinant bacterium enters the external environment without causing any environmental impact. Therefore, the environmentally friendly recombinant *L. plantarum* vaccine could be used as a safe live vector vaccine to prevent virus infection.

HA is a polypeptide chain consisting of 548 to 552 amino acid residues that plays key roles in viral adsorption and membrane penetration, stimulates the production of neutralizing antibodies that neutralize the infectivity of the virus, and is the main antigen of influenza virus (28). In addition, during viral replication, intracellular proteases cleave HA into two subunits, HA1 and HA2, and the antigenicity of hemagglutinin is mainly concentrated in HA1 (5). Our previous study on recombinant *L. plantarum* expressing the HA2 antigen triggered protective immunity against H9N2 subtype avian influenza virus in chickens, with a significant increase in T cell responses following oral inoculation with NC8-pSIP409-pgsA’-HA2 (16). Hajam et al. used *Salmonella* to express the HA1 antigen, which has dual functions as a delivery system and as a natural adjuvant, that could trigger specific humoral and cell-mediated immune responses (29). Kamble et al. showed that intracellular delivery of HA1 subunit antigens by attenuated *Salmonella* protected chickens from infection with a low-pathogenicity H5N3 subtype virus and increased systemic viral clearance (30). Our results in the present study are consistent with those reported in other studies; specifically, recombinant *Lactobacillus* expressing HA1 can induce specific humoral and cellular immunity, indicating that the HA1 antigen can be used as a target-binding antigen for vector delivery with good immunogenicity.

Previous studies have shown that fusion proteins consisting of a DCpep and protective antigens efficiently delivered by *Lactobacillus* enhance antigen-induced systemic immune responses (31). Recombinant *Enterococcus faecalis* fused to the Eimeria 3-1E protein with a dendritic cell-targeting peptide increased secretory IgA levels in cecal lavage fluid and the proportions of CD4^+^ and CD8^+^ cells in the peripheral blood (32). Recombinant *Lactobacillus casei* expressing a fusion molecule containing dendritic cell-targeting peptides enhanced T helper cell responses in piglets, promoted lymphocyte proliferation and effectively protected piglets from PEDV infection (33). In addition, in our previous study, the mucosal vaccine NC8-pSIP409-HA-DCpep, a recombinant NC8 strain expressing HA and DCpep, was constructed to elicit high serum titers of hemagglutination inhibition (HI) antibodies in mice and induce a robust T cell immune response against H9N2 subtype virus infection in both mouse models and chicken models (34). The results of the present study are consistent with previous studies in which NC8Δ-pWCF-HA1-DCpep exhibited a higher degree of DCs activation while significantly elevating IFN-γ expression within CD4^+^ and CD8^+^ T cells in the MLNs and spleen. Interestingly, HA1-DCpep stimulation did not result in a more pronounced CD4^+^ or CD8^+^ T cell proliferative response in the MLNs, whereas the increase was significant in the spleen, suggesting a dominant T cell response in the spleen during AIV infection.

Conventional vaccines do not protect against influenza virus infection on mucosal surfaces, and IgA is one of the major immune effector products present in the gut and plays an important role in preventing natural infection (35). Our previous study showed that recombinant *L. plantarum* NC8 expressing the influenza virus fusion genes HA2 and 3M2e significantly stimulated intestine-specific IgA titers in mice and increased B220^+^IgA^+^ cell numbers in PPs (26). The use of recombinant *Lactobacillus* in other viral infections also elevated the immune response, and oral administration of *Lactobacillus lactis* expressing the HPV-16 L1 antigen in mice induced significant levels of mucosal IgA antibodies (36). This is consistent with our findings that recombinant *L. plantarum* not only elevated the number of B220^+^IgA^+^ cells in PPs and the expression of IgA in different intestinal segments but also induced higher levels of antibody production in lung infection sites. Furthermore, sIgA antibodies showed cross-protection against variant influenza viruses in a mouse model (37). Thus, a mucosal influenza vaccine that induces mucosal immunity would be a powerful tool to protect individuals against influenza viruses.

In summary, *L. plantarum* displaying the influenza virus antigen HA1-DCpep on its surface modulates the activation state of dendritic cells to improve DC function and enhance T cell responses and proliferation. We also demonstrated that recombinant *L. plantarum* increased IgG1, IgG2a and IgA levels and improved serum HI levels. This study demonstrates that HA1-DCpep-expressing recombinant *L. plantarum* can effectively exert immune effects against influenza viruses and has potential as a mucosal vaccine.

## Data Availability Statement

The original contributions presented in the study are included in the article/**Supplementary Material**. Further inquiries can be directed to the corresponding authors.

## Ethics Statement

The animal study was reviewed and approved by Experimental Animal Centre of Jilin Agricultural University (JLAU20210423001).

## Author Contributions

W-TY, G-LY, and C-FW contributed to the conception of the study. HN, J-HX, and B-SZ contributed significantly to analysis and manuscript preparation. C-WS, H-BH, Y-LJ, and J-ZW performed the data analyses and wrote the manuscript. XC, NW, and YZ helped perform the analysis with constructive discussions. All authors contributed to the article and approved the submitted version.

## Funding

This work has supported by the National Natural Science Foundation of China (31972696, 31941018, 32072888), the Science and Technology Development Program of Jilin Province (20210202102NC, 20180201040NY, 20190301042NY, YDZJ202102CXJD029), the Supported by China Agriculture Research System of MOF and MARA, the Key Funds for Agriculture Ministry Key Laboratory of Healthy Freshwater Aquaculture (ZJK201808), the State Key Laboratory of Veterinary Biotechnology Foundation (SKLVBF201909).

## Conflict of Interest

The authors declare that the research was conducted in the absence of any commercial or financial relationships that could be construed as a potential conflict of interest.

## Publisher’s Note

All claims expressed in this article are solely those of the authors and do not necessarily represent those of their affiliated organizations, or those of the publisher, the editors and the reviewers. Any product that may be evaluated in this article, or claim that may be made by its manufacturer, is not guaranteed or endorsed by the publisher.
