# Supplementary material for: Immune Evaluation of Recombinant Lactobacillus plantarum With Surface Display of HA1-DCpep in Mice
Source: Front Immunol. 2021 Dec 1;12:800965. doi: 10.3389/fimmu.2021.800965 (PMC8673267; doi:10.3389/fimmu.2021.800965)
Supplement: Supplementary file 1 [file DataSheet_1.docx]

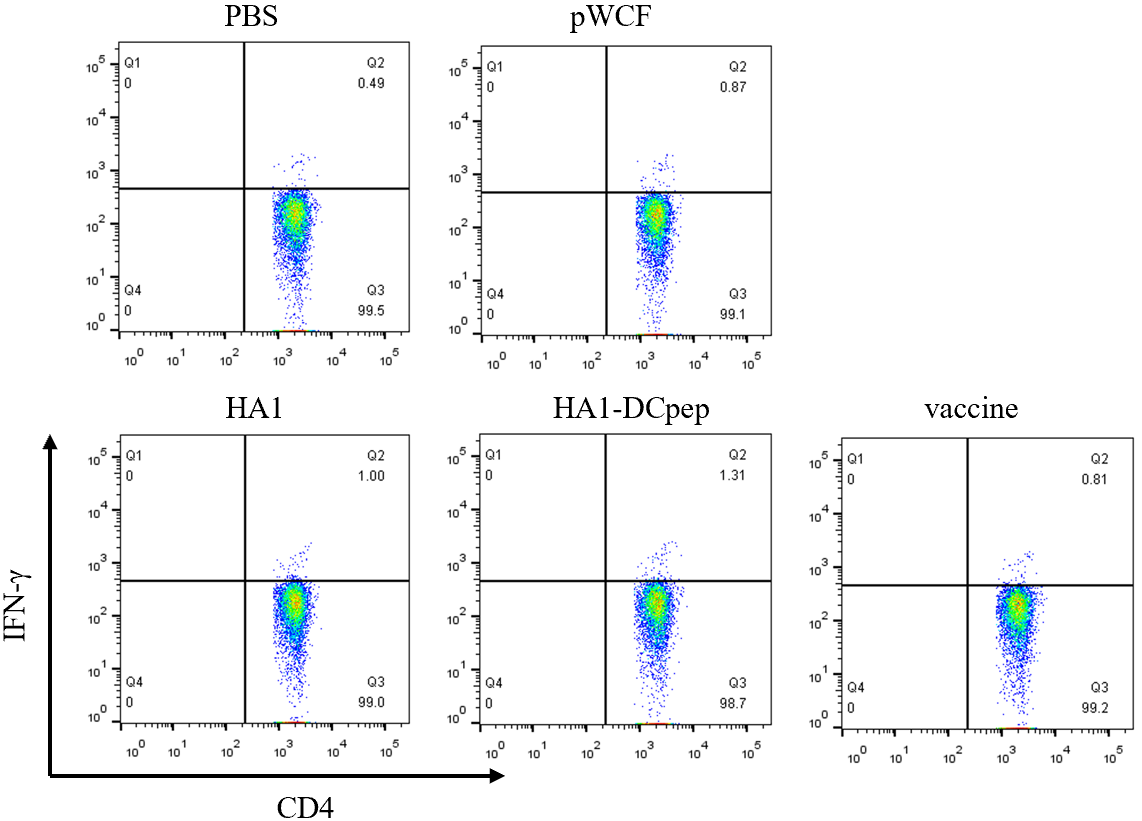

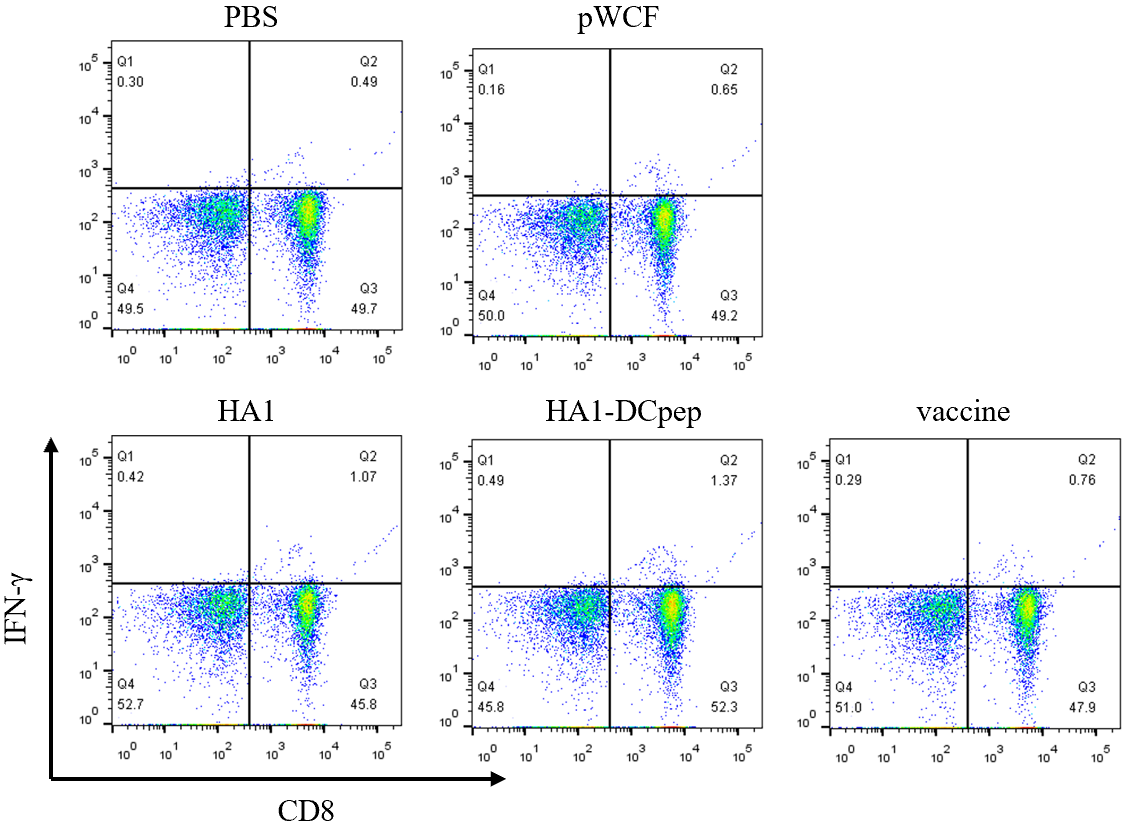

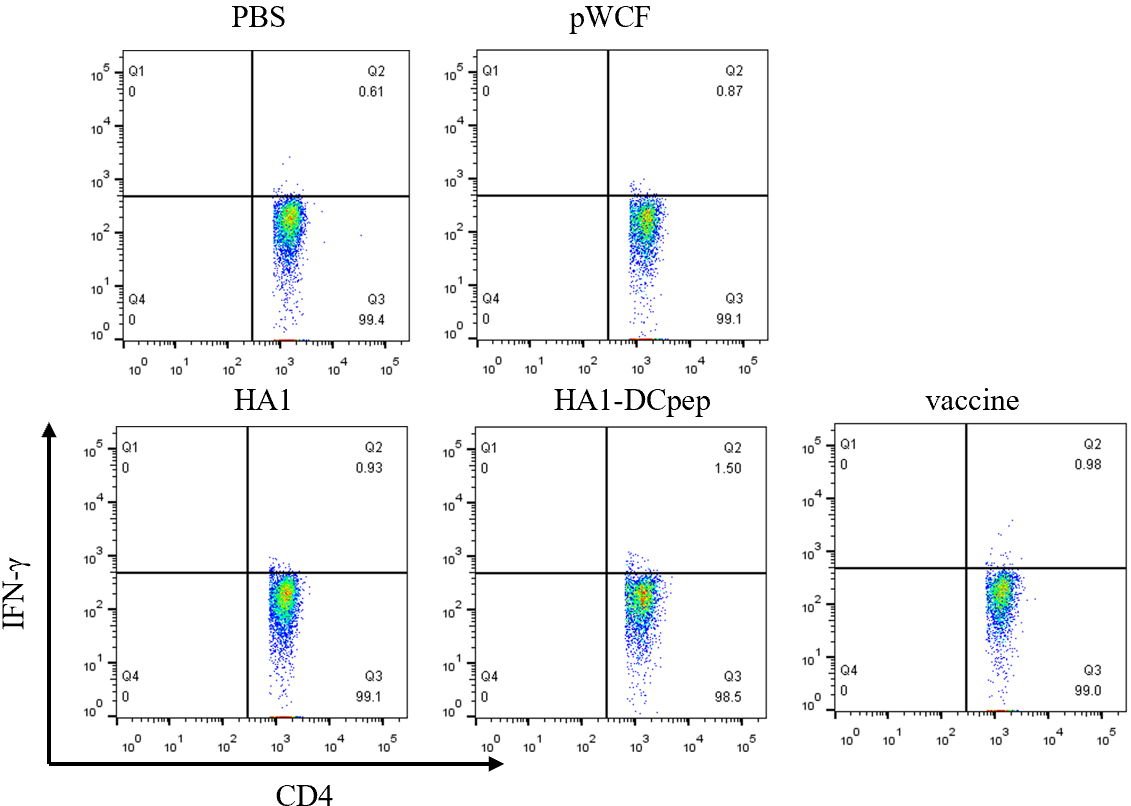


a

b

c

d


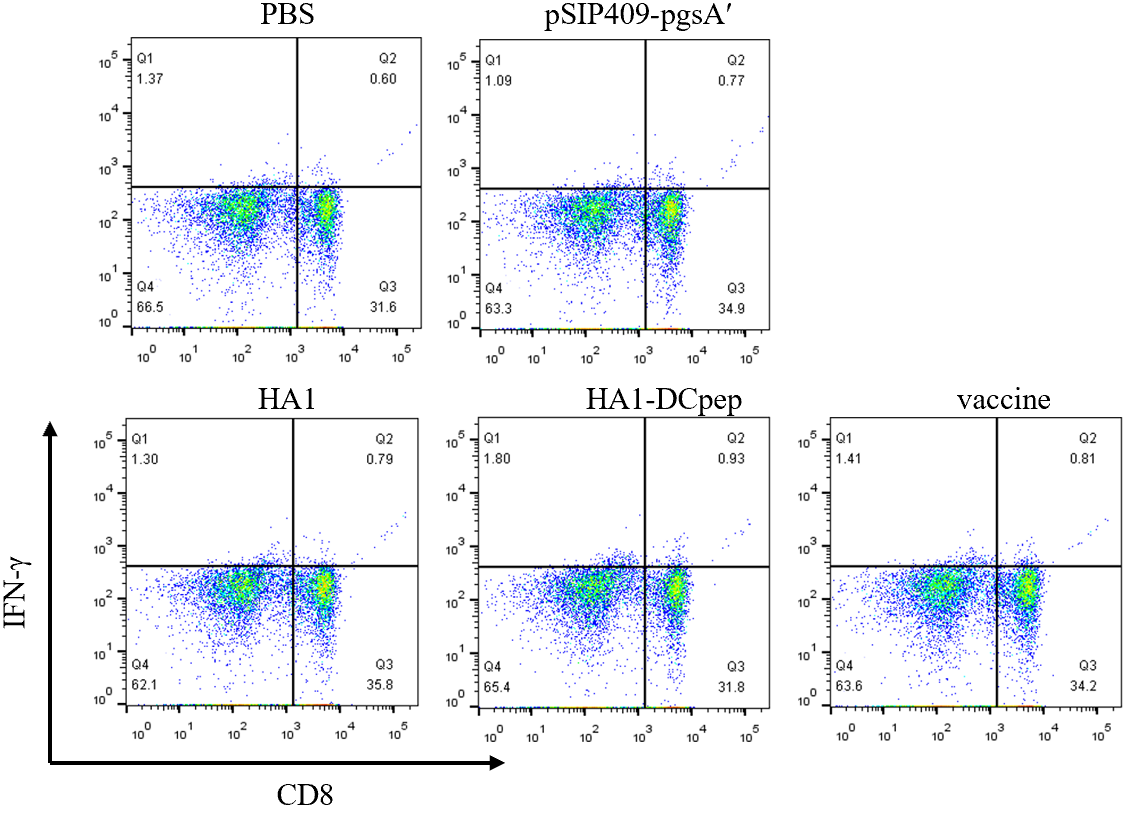

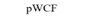


Fig. S1**.** Effect of recombinant *L. plantarum* on T cell responses (flow cytometry scatter plot analysis). After booster immunization, mice MLNs and spleens were collected, and cell suspensions was prepared. Plates were seeded using a total of 1.5×10^6^ cells and incubated with PMA and specific antigenic peptides for 8 hours. The numbers of CD4^+^IFN-γ^+^ T cells (a) and CD8^+^IFN-γ^+^ T cells (b) in the MLNs and the numbers of CD4^+^IFN-γ^+^ T cells (c) and CD8^+^IFN-γ^+^ T cells (d) in the spleen were detected by flow cytometry after antibody staining.


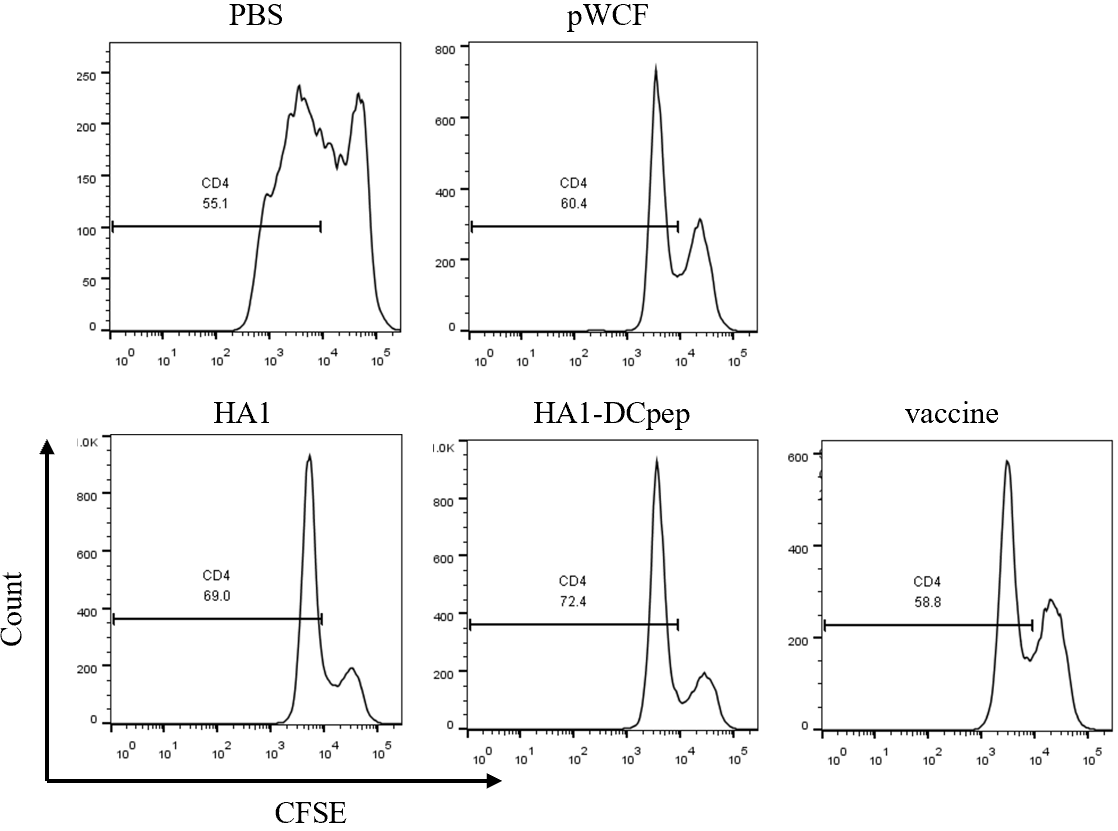

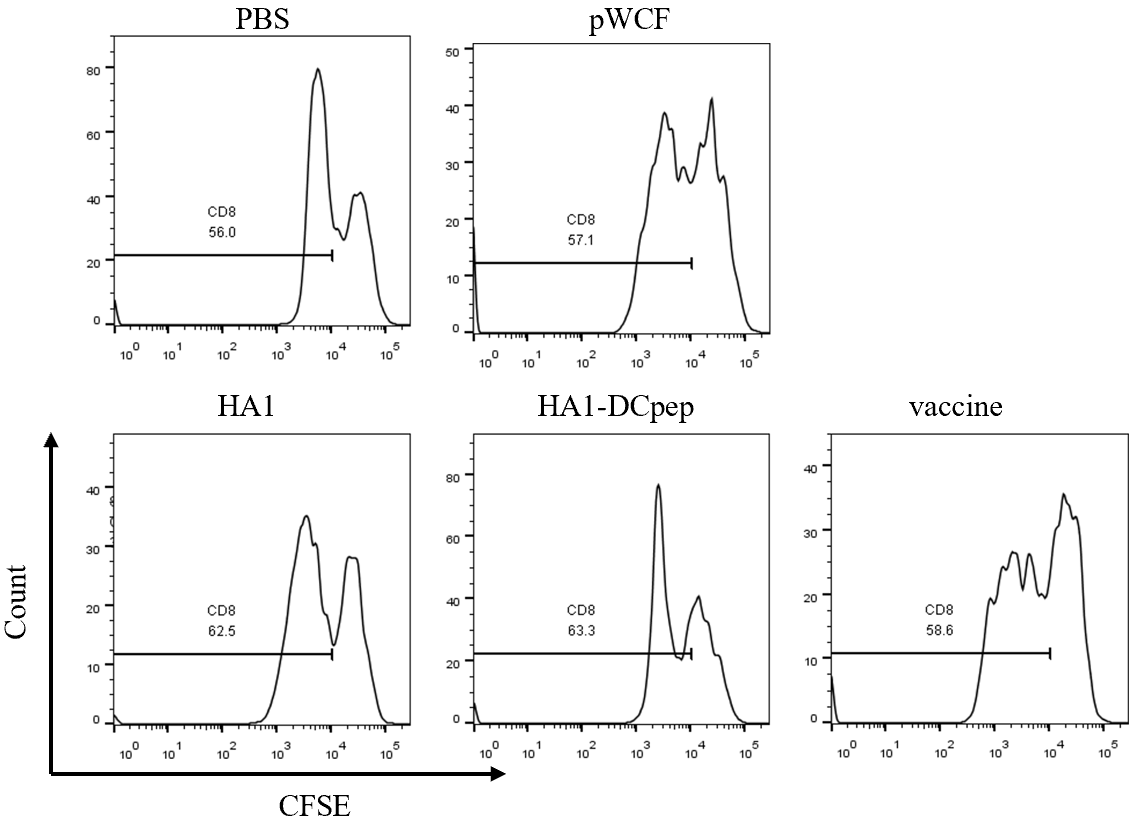

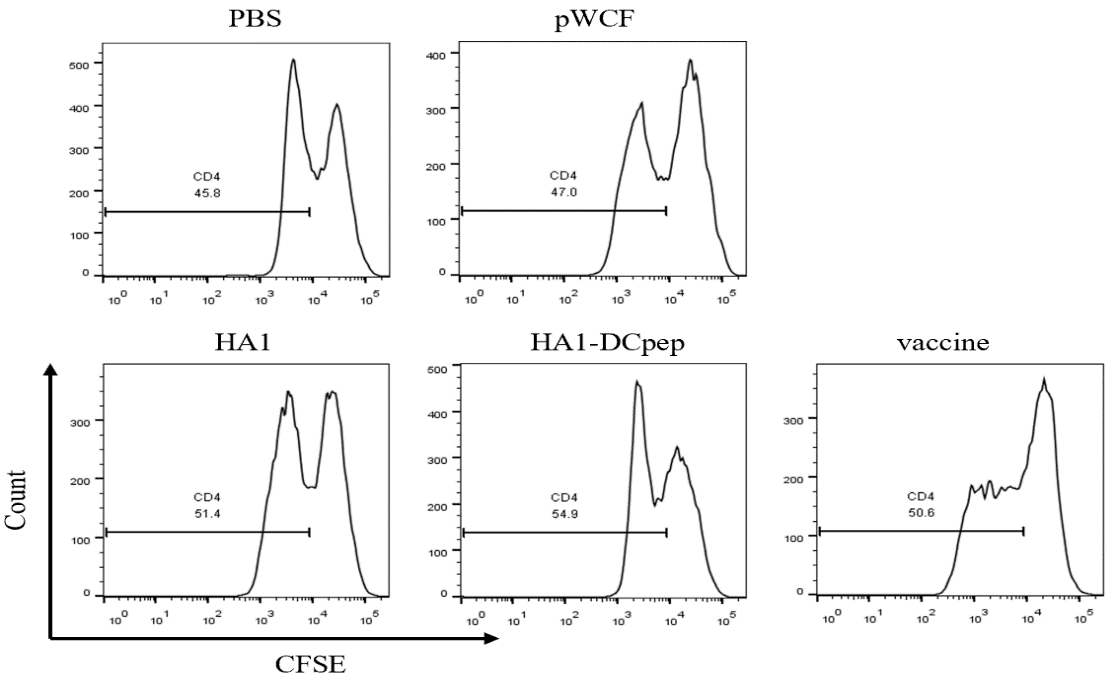


a

b

c


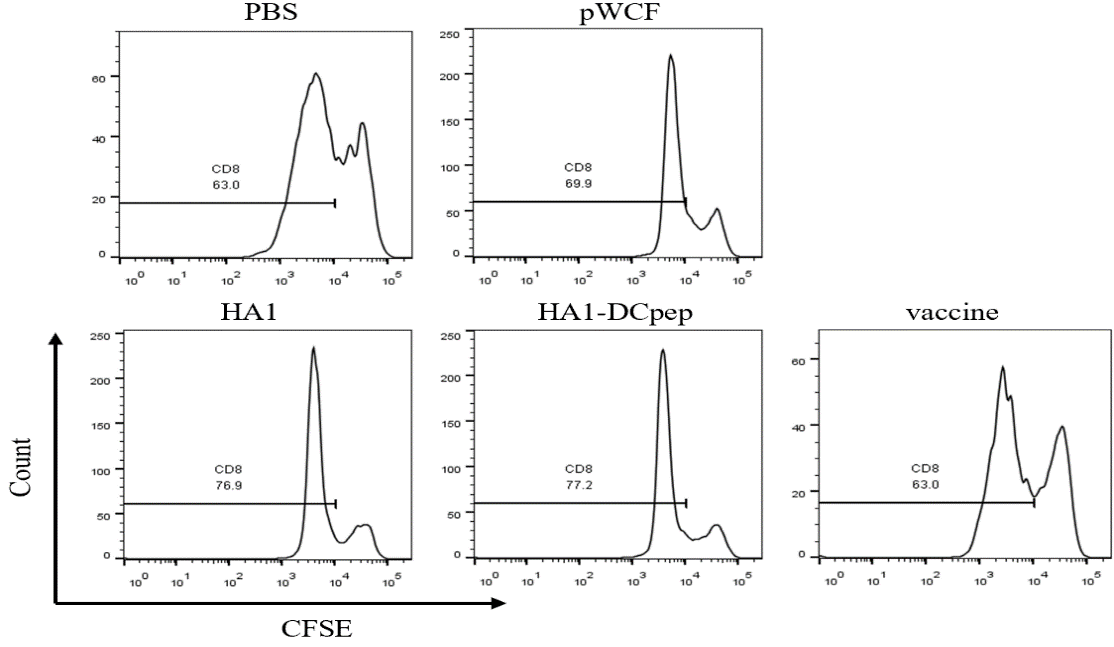


d

Fig. S2. Effect of recombinant *L. plantarum* on T cell proliferation (flow cytometry quantitative statistical analysis). After booster immunization, mice MLNs cells and splenocytes were stained with CFSE. Cells were then placed in 96-well U-bottom plates and cocultured for 3 days using specific antigenic peptides. Flow cytometry was performed to detect the proliferation of CD4^+^ T cells (a) and CD8^+^ T cells (b) in the mice MLNs and CD4^+^ T cells (c) and CD8^+^ T cells (d) in the spleen.
